# Sustaining weight loss among adults with obesity using a digital meal planning and food purchasing platform for 12, 24, and 36 months: a longitudinal study

**DOI:** 10.1186/s12937-021-00666-9

**Published:** 2021-01-21

**Authors:** Emily A. Hu, Mahesh Pasupuleti, Viet Nguyen, Jason Langheier, Dexter Shurney

**Affiliations:** 1 Zipongo, Inc, DBA Foodsmart, 595 Pacific Ave, 4th Floor, San Francisco, CA 94133 USA; 2 Adventist Health, 1 Adventist Health Way, Roseville, CA 95661 USA

**Keywords:** Obesity, Digital, Nutrition, Meal planning, Weight loss, Weight maintenance, Sustained, Dietary score, Mobile app

## Abstract

**Background:**

Previous studies have shown that lifestyle changes, such as diet and exercise, can lead to weight loss, resulting in dramatic improvements in overall health and chronic disease risk. However, while many traditional dieting, food tracking and weight loss coaching programs result in short-term weight loss, there is less evidence of their effectiveness on sustaining weight loss over time.

**Methods:**

We conducted a retrospective analysis of 1,740 adults with obesity who used Foodsmart, a digital personalized dietary assessment, meal planning and food purchasing platform. Participants reported age, gender, at least three measures of weight, and their diet using a food frequency questionnaire. We defined sustained weight loss as participants who lost 5 % of initial weight between their first and second reported weights and lost weight or maintained weight between second and third reported weights. A healthy eating score, Nutriscore, was calculated to assess overall diet quality. We used multivariate logistic regression models to examine the association between user characteristics and odds of sustained weight loss.

**Results:**

Over a median of 25 months, the mean (standard deviation) change in weight among participants was − 6.2 (19.8) pounds. In total, 39.3 % (684/1,740) of participants lost at least 5 % of their initial weight, and 22.4 % percent (389/1,740) of participants sustained weight loss. In the fully-adjusted logistic regression model, we found that obesity class 2 (odds ratio, OR: 1.69, 95 % confidence interval, CI: 1.27–2.24, *P* < 0.001), obesity class 3 (OR: 2.23, 95 % CI: 1.68–2.97, *P* < 0.001), baseline diet quality (OR: 1.06, 95 % CI: 1.02–1.09, *P* < 0.001), and greater change in diet quality (OR: 1.10, 95 % CI: 1.07–1.14, *P* < 0.001) were significantly associated with sustained weight loss.

**Conclusions:**

This study characterized and demonstrated the utility of Foodsmart, a digital platform that gives personalized nutrition recommendations and meal planning tools, in sustained weight reduction among users with obesity.

## Background

Obesity is a growing public health and economic burden as it is a leading cause of chronic illnesses and mortality, resulting in extraordinary healthcare costs [1–3]. While prevention of obesity and weight gain is crucial to reducing the incidence of serious chronic illnesses, efforts to reverse obesity are just as urgent as the prevalence of obesity increases [4, 5]. Among people with obesity, losing weight and maintaining a healthy weight can prevent future comorbidities and health complications [6].

Changes in lifestyle behaviors such as diet and physical activity are frequently used in weight loss interventions [7]. Large behavior change trials such as the Diabetes Prevention Program (DPP) have shown that changes in lifestyle can have dramatic effects on health and chronic disease, often related to weight reduction [8, 9]. However, frequently people who lose weight from lifestyle interventions like DPP regain weight after 12 months, which can have adverse health consequences and be more costly due to repeatedly returning to weight loss programs [10, 11]. Weight cycling may put additional stress on the cardiovascular system through negative effects on blood pressure levels, heart rate, sympathetic activity, glucose, lipids, and insulin [11].

Digital technologies have been incorporated into obesity prevention and weight loss strategies as they have become integrated into everyday life for many individuals [12, 13]. While many diet tracking and nutrition education mobile applications have been successful in achieving weight loss among individuals with overweight or obesity, there is less evidence on how successful they are in maintaining weight loss [14].

Furthermore, the majority of commercial nutrition mobile apps focus on nutrition education, coaching, or diet monitoring and logging [15]. However, a multi-pronged approach that addresses an individual’s environment to break down barriers to healthy eating through knowledge, access, and cost may be more successful in creating lasting behavior change. In addition to helping educate and track an individual’s dietary and weight progress, altering the food purchasing and cooking environment is a macro-level change that can facilitate healthier choices in grocery shopping and meal planning. Brick-and-mortar grocery stores, from supermarkets to convenience stores, are subject to imbalanced advertising and product placement of unhealthy foods, leading to impulse purchases [16]. The removal of advertising of these tempting processed foods in an online grocery ordering setting and nudges towards healthier foods may change the environment to positively influence the user to make healthier decisions. Previous studies have shown that higher frequency of cooking and eating at home is associated with healthier diet quality, fewer calories consumed, and greater weight loss [17–20].

Given the paucity of studies examining the sustainability of weight loss after more than 12 months, the aim of this study was to examine the effectiveness of a digital nutrition, meal planning, and food purchasing tool in weight loss after 12, 24, and 36 months among users with obesity.

## Methods

### Foodsmart

**Fig. 1:**
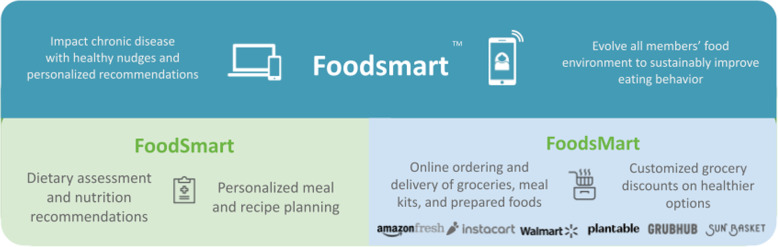
Components of Foodsmart

Foodsmart™ is a digital nutrition platform that encourages lasting behavior change through personalization of nutrition and meal/recipe recommendations and through altering the food purchasing environment to provide healthy eating options. The two main components are FoodSmart and FoodsMart, which both use behavior change theory to facilitate access and engagement with affordable, tasty, and healthy food (Fig. 1).

The FoodSmart component contains the in-app Nutriquiz, a food frequency questionnaire (based on the National Cancer Institute’s Diet History Questionnaire) which users take to report their dietary habits, and that provides immediate feedback on areas they can improve upon as well as personalized meal and recipe planning based on the Nutriquiz results. The Nutriquiz also ascertains demographic information, weight, and clinical conditions. The user can retake the Nutriquiz at any time, allowing them to monitor their progress on diet and weight. The other component is FoodsMart, which helps alter the user’s food purchasing environment through personalized meal planning. Users can add to their grocery list within the app and then use integrated online ordering and delivery of groceries, meal kits, and prepared foods. Customized grocery discounts on healthier options help the user save money and further nudges the user to make healthier choices.

Foodsmart is available through certain health plans and employers, who provide this product as an option or benefit for their members/employees to enroll in. It is available on web, iOS, and Android.

### Study population

We conducted a longitudinal, retrospective analysis of 1,740 adults with obesity living in the U.S. who used Foodsmart between January 2013 and April 2020. As of April 2020, there were 888,999 users who had signed up for Foodsmart. Among all of the users, we excluded individuals who did not report weight (*n* = 562,276), individuals who reported extreme values for height (< 54 or > 78 inches) or weight (< 60 or > 400 pounds) (*n* = 25,946), and individuals who did not have obesity [body mass index (BMI) < 30 kg/m^2^] at first weight entry (*n* = 200,308). We further excluded individuals who did not report weight at least three times and participants with less than 1 month between first and second or second and third weight report (*n* = 98,729). The final analytic sample included 1,740 users.

### Dietary Assessment

Dietary data were self-reported through Foodsmart. Upon registration, users were prompted to fill out a dietary questionnaire called “Nutriquiz”, a 53-item food frequency questionnaire adapted from the National Cancer Institute Diet History Questionnaire, which has been previously validated [21]. Information on sex, age, weight, and usual frequency of dietary intake (fruits, vegetables, whole grains, proteins, carbohydrates, fats, fiber, sodium, and water) are collected through the Nutriquiz. A healthy diet score created by the Foodsmart research team called Nutriscore was calculated, which is derived from the Alternative Healthy Eating Index-2010, a previously validated score among several U.S. cohorts, and the Commonwealth Scientific and Industrial Research Organization (CSIRO) Healthy Diet Score [22, 23]. Participants were assigned a score from 0 to 10 (with 10 being optimal) for each of seven components: fruits, vegetables (excluding potatoes), protein ratio (white meat/vegetarian protein to red/processed meat), carbohydrate ratio (total fiber to total carbohydrate), fat ratio (polyunsaturated to saturated/trans fat), sodium, and hydration (percent of daily fluid goal). A total Nutriscore (possible scores ranging from 0 to 70) was calculated by summing the scores of the seven components. Change in Nutriscore was calculated as the difference between a participant’s first and last Nutriscores. We categorized participants by whether their Nutriscore decreased or was stable (no improvement in diet quality) versus increased (improvement in diet quality).

### Measurement of Weight

Users were given the option to add weight and height data when they first created their Foodsmart account and could update their weight at any time during usage of the platform. Baseline BMI was calculated as first weight entry in kilograms divided by height in meters squared (kg/m^2^). We categorized participants by baseline obesity class. Class 1 obesity was defined as a BMI between 30 and 34.9 kg/m^2^, class 2 was defined as a BMI of 35 to 39.9 kg/m^2^, and class 3 was defined as a BMI of 40 kg/m^2^ or higher.

Our primary outcome was sustained weight loss, which we defined as losing 5 % of initial weight between first and second reported weights and additional weight loss or no change between the second and third reported weights.

Duration of enrollment (in months) in Foodsmart was calculated as the number of months between the first activity date and last activity date.

### Statistical analysis

We used descriptive analyses to examine baseline characteristics of the total study population and by whether participants sustained weight loss or not. We reported categorical variables as frequencies (%) and continuous variables as mean ± standard deviation (SD).

To investigate long-term efficacy of Foodsmart on sustained weight loss, we examined the percent of participants who sustained weight loss by the duration of their enrollment time (by 12, 24, and 36 months). Further, we examined the percent of participants by each category of age, baseline obesity class, and change in Nutriscore. We used chi-square tests to determine whether differences within each category were statistically significantly different.

Multivariate logistic regression models were used to estimate odds ratios (OR) and 95 % confidence intervals (CI) of sustained weight loss adjusted for gender, age category, baseline obesity category, baseline Nutriscore (per 2-point increase), and change in Nutriscore (per 2-point increase).

We considered a *P*-value smaller than 0.05 to be significant for all tests. Stata version 16 was used for all analyses (StataCorp, College Station, Texas).

The study was declared exempt from Institutional Review Board oversight by the Pearl Institutional Review Board given the retrospective design of the study and less than minimal risk to participants.

## Results

### Participant characteristics

Baseline demographic characteristics and weight metrics of the total study sample and stratified by whether participants had sustained weight loss are shown in Table 1.

**Table 1:** Baseline characteristics of all participants and by sustained weight loss

	Total (*n* = 1,740)	Did not sustain weight loss (*n* = 1,351)	Sustained weight loss (*n* = 389)
Age, yrs	48 ± 11	48 ± 11	49 ± 11
Male, %	16 %	16 %	18 %
Height, inches	66 ± 3	66 ± 3	66 ± 3
Baseline weight, lbs	225 ± 40	222 ± 39	235 ± 42
Baseline BMI, kg/m^2^	37 ± 6	36 ± 6	38 ± 6
Obesity class			
Obesity class 1 (BMI 30.1–35 kg/m^2^)	51 %	54 %	39 %
Obesity class 2 (BMI 35.1–40 kg/m^2^)	25 %	25 %	28 %
Obesity class 3 (BMI > 40.1 kg/m^2^)	24 %	21 %	33 %
Baseline Nutriscore	30.3 ± 8.5	30.3 ± 8.6	30.5 ± 8.5
Final Nutriscore	33.7 ± 8.6	33.1 ± 8.6	35.7 ± 7.9
Change in Nutriscore	3.3 ± 7.9	2.8 ± 7.8	5.2 ± 8.0
Enrollment length, months	25 ± 10	25 ± 10	25 ± 10
Weight change, %	-2.5 ± 8.6	0.2 ± 7.0	-12.1 ± 6.6
Weight change, lbs	-6.2 ± 19.8	0.3 ± 15.4	-28.5 ± 17.2
Weight change from 1st to 2nd report, lbs	-4.0 ± 15.6	-0.1 ± 13.7	-17.6 ± 14.4
Weight change from 2nd to 3rd report, lbs	-2.1 ± 14.2	0.4 ± 14.4	-10.9 ± 9.2

Categorical variables were reported as frequencies (%) and continuous variables were reported as mean ± standard deviation (SD)

Abbreviations: *BMI* body mass index; *kg* kilograms; *lbs* pounds; *m* meter

There were 1,740 participants included in the analysis, of which the mean age was 48 years and 16 % were male (Table 1). The mean and median enrollment length was 25 months. In total, 39.3 % (684/1,740) of participants lost at least 5 % of their initial weight in the first time period, and 22.4 % percent (389/1,740) of participants sustained weight loss. Compared to participants who did not sustain weight loss, participants who did sustain weight loss were more likely to have been categorized in a higher obesity class at baseline, have a higher change in Nutriscore, and experienced greater weight loss.

**Fig. 2:**
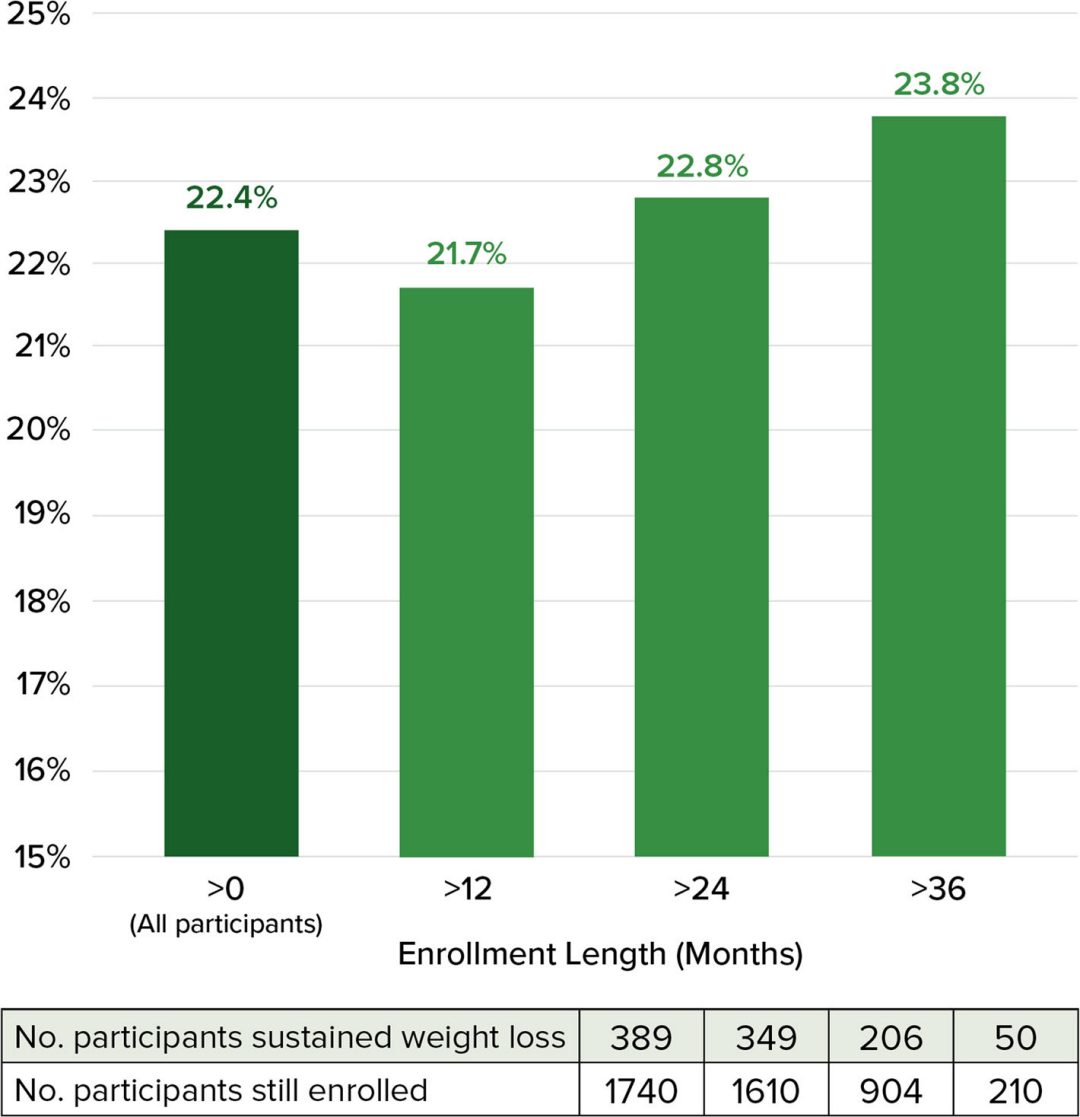
Percent of participants who sustained weight loss by cumulative enrollment time

We examined the percent of participants who sustained weight loss by cumulative enrollment time in Fig. 2. Among all participants, 22.4 % sustained weight loss. Among participants who were enrolled for greater than 12, 24, and 36 months, the percent of participants who sustained weight loss was, respectively, 21.7 %, 22.8 %, and 23.8 %.

**Fig. 3:**
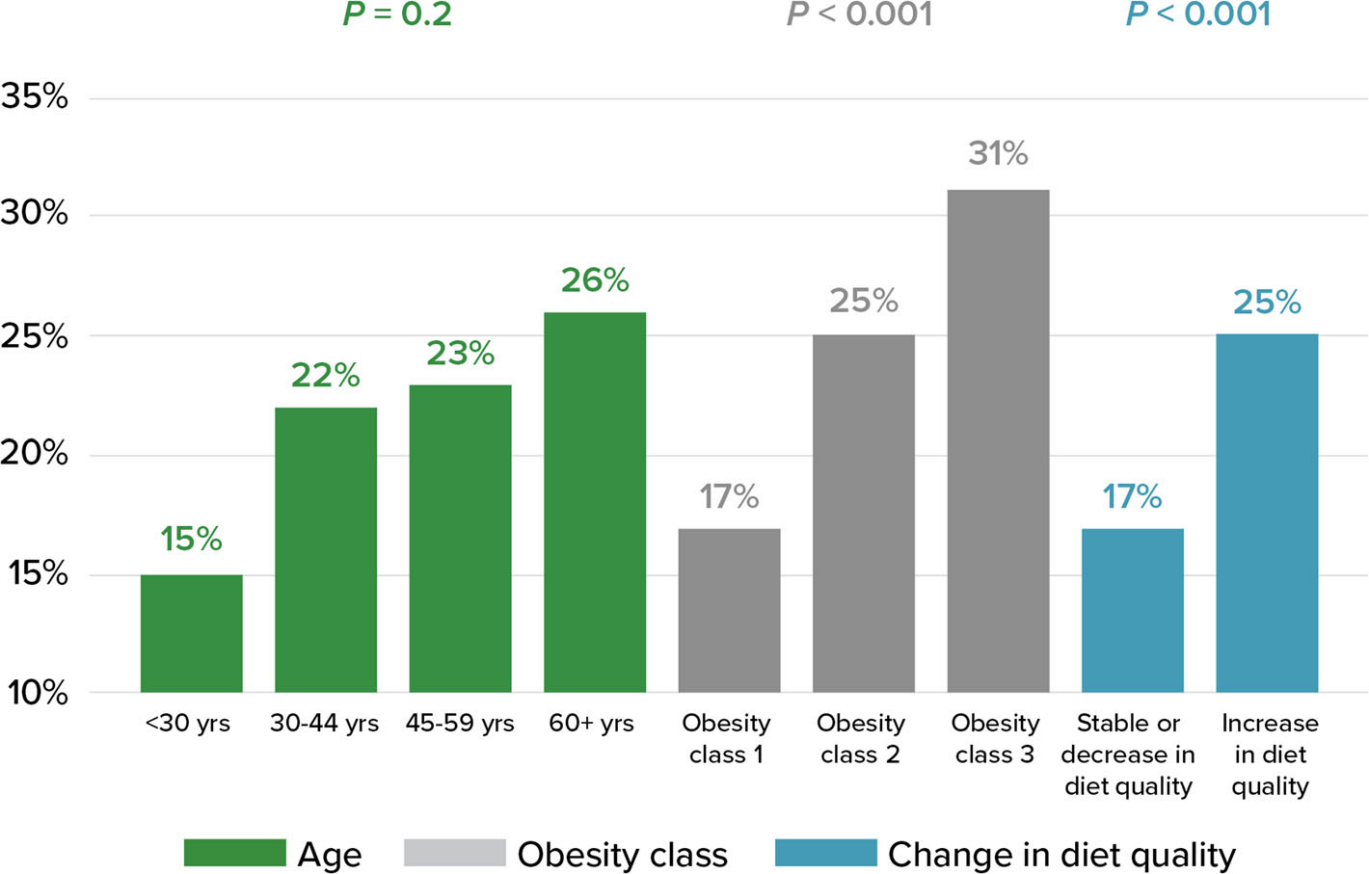
Percent of participants who sustained weight loss by category of age, obesity class, and change in diet quality^1^ ^1^ Chi-square tests were used to test for significant differences between groups within each category

We also examined the percentage of participants who sustained weight loss by categories of age, baseline obesity class, and improvement in Nutriscore (Fig. 3). The percent of participants who sustained weight loss increased with age, baseline obesity class, and improvement in diet quality, however was only statistically significant for the latter two using chi-square tests (*P* < 0.05).

### Predictors of Sustained Weight Loss

We examined predictors of sustained weight loss in multivariate logistic regression (Table 2).

**Table 2:** Factors contributing to sustained weight loss in multivariate logistic regression models

	Multivariate OR (95 % CI)	*P*
Male	1.33 (0.98–1.81)	0.1
Age	1.01 (1.00-1.02)	0.2
Baseline BMI		
Obesity class 1	1 (ref.)	
Obesity class 2	1.69 (1.27–2.24)	< 0.001
Obesity class 3	2.23 (1.68–2.97)	< 0.001
Baseline Nutriscore (per 2 pt increase)	1.06 (1.02–1.09)	< 0.001
Change in Nutriscore (per 2 pt increase)	1.10 (1.07–1.14)	< 0.001

Abbreviations: *BMI* body mass index; *CI* confidence interval; *kg* kilogram; *m* meter; *OR* odds ratio; *ref* reference; *yrs* years

There was no significant association between gender and sustained weight loss (*P* = 0.1) or age and sustained weight loss (*P* = 0.2). Compared with individuals who were in obesity class 1 at baseline, those who were in obesity class 2 and obesity class 3 had a 69 % and 123 %, respectively, increased likelihood of sustained weight loss. Each additional two-point increase in baseline Nutriscore was associated with a 6 % increased likelihood of sustained weight loss (OR: 1.06, 95 % CI: 1.02–1.09, *P* < 0.001) and each two-point increase in change in Nutriscore was associated with 10 % increased likelihood of sustained weight loss (OR: 1.10, 95 % CI: 1.07–1.14, *P* < 0.001).

## Discussion

We found that among 1,740 adults with obesity who used Foodsmart, a digital meal planning and food purchasing platform, 22.4 % of participants sustained weight loss (lost 5 % of initial weight and then maintained the same weight or lost more weight). Sustained weight loss was observed consistently after 12 (21.7 %), 24 (22.8 %), and 36 (23.8 %) months of engagement with the platform. Baseline obesity class, baseline Nutriscore, and change in Nutriscore were predictors associated with greater odds of sustained weight loss in multivariate regression models.

Lifestyle interventions to manage and prevent obesity have been demonstrated to be effective in weight loss. Programs such as the DPP lifestyle intervention have been proven to be more effective than pharmacological interventions in preventing incident diabetes and resulted in weight loss [7]. Mobile health interventions have also been found to be successful in the treatment adults with overweight or obesity; however, there is insufficient evidence on their effectiveness for weight loss maintenance [14]. While many mobile nutrition and health apps help users self-monitor their dietary behaviors and health outcomes or health coaching, few actually change the user’s environment to enable long term changes in health behavior [24]. A review of web-based weight loss interventions among adults with overweight or obesity found that the percentage of weight loss ranged from none to 5.8 % over a range of 3 to 18 months [25]. Among the studies that examined weight maintenance, one study found that participants in the Internet intervention lost on average 5.3 kg after the 4 month intervention, but gained weight (0.7 kg) after an additional 12-month follow-up [26]. Another study found that the internet support intervention group lost 8.4 kg over 6 months and after an additional 12-month follow-up, regained 0.8 kg [27].

Major barriers to healthy eating include access to healthy food, time to shop, price, and selection of healthy foods, especially among low income populations [28]. Few interventions are designed to address these aspects in a holistic manner. Foodsmart seeks to remove these barriers by providing an online grocery shopping experience to save time, discounts and deals, and meal planning features for personalized, healthy recipes. This type of model for food purchasing is especially relevant during the COVID-19 pandemic, where grocery shopping in brick-and-mortar stores is discouraged and online shopping orders have increased [29, 30]. The rise in online grocery ordering and food purchasing/delivery may pave a new future for how food is purchased [30].

Foodsmart provides a toolkit of features that are personalized for the user and offers advice at almost every opportunity a user comes in contact with food to help users eat healthier and has been shown to result in weight loss among users with obesity [31]. Grounded in the Theory of Change behavior theory model, the specific features of the platform are intended to target all stages of behavior change (pre-contemplation, contemplation, preparation, action, and maintenance) [32]. The Nutriquiz and meal planning features help target stages of contemplation and preparation. They empower the user to eat healthier by equipping them with the nutritional knowledge of what opportunities there are for improvement in their current diet and recommend recipes personalized to their taste, preparing users to take an active role in shopping and eating healthier. The grocery list, food ordering, rewards, and discounts are designed to change actual shopping and eating behavior. When users recognize that they are saving time and money, eating healthier and feeling better, users are likely to maintain their changes in behavior to continue using the platform and eat healthy. As we found in our analyses, a reported increase in Nutriscore over time was associated with greater odds of sustained weight loss. Greater engagement with the app is likely to improve the diet, as represented by an increase in Nutriscore, which translates to weight loss and sustained weight loss.

The present study has some limitations. This study was observational, without an intervention or control group, and thus we cannot conclude a causal association between the Foodsmart platform and sustained weight loss. However, the study seeks to explore which factors are associated with sustained weight loss among Foodsmart users. All measures of height, weight, and diet were self-reported by participants. However, prior studies suggest that there is moderate to high agreement between online self-reported and measured anthropometric data [33]. Another limitation was that a very small sample of users entered their weight three times, leading to potential selection bias. These users were most likely more health- and weight-conscious and may not be representative of all users. The time between weight entries was not standardized since users could enter their weight at any time. Although we could not control for this, it allowed us to compare sustained weight loss at different time periods, and we found the percent of people with sustained weight loss was consistent at 12, 24, and 36 months. We did not have information on other potentially important predictors of weight loss such as physical activity level, race, or socioeconomic status since this data was not collected in Foodsmart. Additionally, we did not have data on pre-existing diseases or medication use, nor menopausal status, which could play an important role in weight change among females, especially among those with obesity [34].

There were also many strengths in this study. Foodsmart’s database contains about 1 million users’ data related to dietary responses, and a subset of them have weight data. Conducting a formal analysis in a large, real-world dataset like this allows us to leverage the power of technology to gain insights about how users respond to new behavior-change technologies like Foodsmart. While there are many commercial applications that are targeting behavior-change through nutrition or other lifestyle modifications, very rarely do they have the opportunity to examine their data to make statistical associations that help the scientific field better understand interventions and solutions used in the real world. Another strength is that participants were enrolled with Foodsmart for extended periods (up to 55 months), allowing us to examine weight change and maintenance within a time span of more than three years. We also had a large sample size, allowing us to conduct subgroup analyses.

This study found that adults with obesity who used a digital nutrition platform with personalized dietary recommendations and online meal planning, food ordering, grocery discounts and incentives were able to sustain weight loss after 12, 24, and 36-month intervals. Additional research through randomized controlled trials and cost-effectiveness studies are needed to elucidate causal associations and to determine the economic impact of this type of digital technology.

## Conclusions

In conclusion, this study demonstrated the clinical utility of a digital platform that gives personalized nutrition recommendations and meal planning and grocery delivery tools in sustained weight reduction among users with obesity.

## Data Availability

The datasets generated and/or analysed during the current study are not publicly available due to legal contracts with employer and health plan partners but are available from the corresponding author on reasonable request.

## Abbreviations

BMI: Body mass index
CI: Confidence interval
CSIRO: Commonwealth Scientific and Industrial Research Organization
DPP: Diabetes Prevention Program
kg: Kilograms
lbs: Pounds
m: Meter
OR: Odds ratio
SD: Standard deviation
